# Finding toxicological information: An approach for occupational health professionals

**DOI:** 10.1186/1745-6673-3-18

**Published:** 2008-08-13

**Authors:** Irja Laamanen, Jos Verbeek, Giuliano Franco, Marika Lehtola, Marita Luotamo

**Affiliations:** 1 Finnish Institute of Occupational Health, Topeliuksenkatu 41 a A, 00250, Helsinki, Finland; 2 Finnish Institute of Occupational Health, Kuopio, Finland; 3 Kuopio University, Kuopio, Finland; 4 Universita' di Modena e Reggio Emilia Modena, Italy

## Abstract

**Background:**

It can be difficult for occupational health professionals to assess which toxicological databases available on the Internet are the most useful for answering their questions. Therefore we evaluated toxicological databases for their ability to answer practical questions about exposure and prevention. We also propose recommended practices for searching for toxicological properties of chemicals.

**Methods:**

We used a systematic search to find databases available on the Internet. Our criteria for the databases were the following: has a search engine, includes factual information on toxic and hazardous chemicals harmful for human health, and is free of charge. We developed both a qualitative and a quantitative rating method, which was used by four independent assessors to determine appropriateness, the quality of content, and ease of use of the database. Final ratings were based on a consensus of at least two evaluators.

**Results:**

Out of 822 results we found 21 databases that met our inclusion criteria. Out of these 21 databases 14 are administered in the US, five in Europe, one in Australia, and one in Canada. Nine are administered by a governmental organization. No database achieved the maximum score of 27. The databases GESTIS, ESIS, Hazardous Substances Data Bank, TOXNET and NIOSH Pocket Guide to Chemical Hazards all scored more than 20 points. The following approach was developed for occupational health professionals searching for the toxicological properties of chemicals: start with the identity of the chemical; then search for health hazards, exposure route and measurement; next the limit values; and finally look for the preventive measures.

**Conclusion:**

A rating system of toxicological databases to assess their value for occupational health professionals discriminated well between databases in terms of their appropriateness, quality of information, and ease of use. Several American and European databases yielded high scores and provide a valuable source for occupational health professionals.

## Background

Workers are exposed to toxic chemicals in many jobs. For the worker, exposure may constitute a risk, for occupational health professionals (OHPs) a need to respond. In case of exposure, OHPs must find out if the chemicals used in the workplace cause hazard(s), risk(s), symptom(s), and/or diseases. To prevent exposures they need to know the properties of the chemicals used and the relationship between dose or level of exposure to the substance and the severity of the effect.

To answer such questions, an increasing number of databases are currently available on the Internet. However, it is not easy to find databases to cater for the needs of occupational health practitioners because the practical viewpoint is often missing. Articles written about toxicological databases address researchers, unspecified users, health care professionals in general, or persons that require information for a specific purpose. These articles of the databases may begin with a very specific point of view, such as developmental toxicity, or only describe the features of sources [1-4]. Judging whether the sources and information presented are applicable and credible presents a challenge. Rating systems used to assess the validity of contents often lack a satisfactory degree of reliability and validity [5-9].

Several authors have written about barriers in finding and using information. Schaafsma, Bennett and others emphasize that professionals must learn which search engines and sites can be trusted [10-13]. The number of chemical substances and mixtures is myriad, and what is written about their health effects varies and seems largely dependent on the producers of the information. Judging whether the information is applicable and credible may present a great challenge for the end users. The needs also vary according to the role of occupational health professionals. Further, there are certain psychological barriers relating to the information seeker that may prevent the fulfillment of information needs. These include personal preferences, prejudices, self-evaluation of knowledge and skills, interests, and knowledge of the subject and foreign languages. In addition, toxicology has its own terminology and jargon which does not facilitate the challenge for occupational health professionals. Last but not least, there are factors associated with the information itself: whether or not the information is vetted by experts, whether it is generated by an authoritative source and producer, whether it is up-to-date and regularly updated, and whether it can be easily and conveniently accessed [14-16].

Legislative aspects form an essential part of toxicological information. The chemical industry is one of the most regulated of all industries, and chemicals are regulated by legislation all over the world. There is a large body of legislation at the national and international level to ensure and improve the safety and health of the workforce and other users of chemicals. Regulatory bodies for instance in the European Union and in the US produce toxicological profiles of chemicals and make them available via databases. For example, the European REACH (Registration, Evaluation, Authorisation and Restriction of Chemicals) will generate an enormous amount of new toxicological data [17]. Despite of the efforts of governments, it is still today impossible to find a universal database that can provide all toxicological information for all chemicals used in industry [18].

The aim of this article is to provide OHPs with information on how to find and rate the contents, quality and usability of toxicological databases thus generating a model approach for occupational health professionals seeking practical health-related information on chemical substances.

## Methods

We used a 4-step process to answer our research question. First we had to find the databases and select the relevant ones, then develop methods to evaluate their contents, and the third step was the actual rating of contents. Finally, we built a model for the search process.

To create a list of relevant databases accessible in the Internet, we conducted a systematic search on 7 February 2007 with the advanced Google search engine. We used the following search strategy: with all of the words: 'occupational health'; with the exact phrase: 'database'; with at least one of the words: 'toxic' 'hazardous' or 'chemicals '. The search was limited by language: 'English' and by date: 'past year'. The result of the search was over one million web pages out of which the first 822 met all our criteria.

These 822 web pages were screened to find out if they contained a database that fulfilled all of the following criteria: 1) includes a search engine (integrated search engines also accepted), 2) contains factual information on toxic and hazardous chemicals harmful for human health, 3) is free of charge.

We developed a rating scale with three main categories: (i) user needs, (ii) quality of information, and (iii) ease of use. The *user needs *were defined based on the following idea of practical needs of occupational health professionals. The first step is to find the chemical name of the substance. The next central piece of information for the OHP concerns the health hazards involved. In order to evaluate the health risks, the OHPs need information on exposure assessment and evaluation and exposure routes. To be able to take preventive measures, information on what measures are available and effective is needed.

To assess the *quality *of the databases, we used the following indicators: a) topics covered, b) level of information (from peer review to general), c) number of chemicals covered, d) regularity of updates.

For assessment of *usability*, the following indicators were used: a) navigation, b) availability of help, and c) different languages. The exact criteria and their ratings are given in the Table 3.

**Table 1:** List of databases that fulfilled the requirements

**Name of database**	**Country/Organization**	**Internet address**
1. ATSDR-HazDat database	USA	
2. Chemical Sampling Information (CSI)	USA	
3. ESIS – European Chemical Substances Information System	EU	
4. EXTOXNET	USA	
5. GESTIS-database on hazardous substances	GER	
6. Haz-Map	USA	
7. High Production Volume Information System (HPVIS)	USA	
8. Hazardous Substances Data Bank (HSDB)	USA	
9. Hazardous Substances Information System (HSIS)	AUS	
10. IARC Monographs	UN	
11. IPCS INCHEM	UN	
12. International Toxicity Estimates for Risk Database	USA	
13. IRIS database for risk assessment	USA	
14. MSDS Database – (Material Safety Data Sheet db)	CAN	
15. NIOSH Pocket Guide to Chemical Hazards	USA	
16. PAN Pesticides Database	USA	
17. Scorecard	USA	
18. Screening Information Data Set (SIDS) for High Volume Chemicals	UN	
19. SOLV-DB	USA	
20. The chemical database	USA	
21. TOXNET	USA	

Specific criteria were developed for all indicators to enable a more accurate rating concerning the fulfilling of user needs, higher quality, and ease of use.

Four raters with different academic backgrounds (initials and expertise between brackets) assessed the databases independently according to the items mentioned above. They used the following chemicals for their assessments: styrene, amitrole or aminotriazole (IL, biologist, information specialist), aniline (MLu, chemist), lead or formaldehyde (ML, biochemist, and engineer), and formaldehyde (JV, physician). In cases where the first three persons had three different results, the fourth evaluator completed the rating. The final rating presented in the tables is based on agreement of at least two raters.

## Results

We found 21 databases that fulfilled the criteria of providing valuable information on chemical substances free of charge for all users (Table 1). The most common reason for rejecting the databases was that the database did not contain factual information but referred to books or general sources.

Of the databases included in table 1, 14 are administered in the US, five in Europe, one in Australia, and one in Canada. Nine are administered by governmental organizations, four by international or regional organizations, and the remaining eight by non-profit organizations or educational institutes. Six specialize in one category of chemicals, while the rest aim to cover all types of chemicals.

Table 1. List of databases that fulfilled the requirements

Table 2 presents a comparison of the summarized results for the database rating. The judgement of the governmental databases was easier than those from other sources. In general there was more information on the first. Detailed ratings are given in Table 4. The total score for *user needs *ranged from 3 to 8, with none of the databases achieving the maximum score of 9 points. The item most often missing was information on preventive measures, indicating that many of the databases do not entirely meet the needs of OHPs. The raters used different chemicals to account for different users with varying needs. This caused some disagreement among the evaluators and resulted in some variations in the results.

**Table 2:** Comparison of the rating results of needs, quality, usability and total rating.

**Name of database**	User Needs	Quality	Usability	Total Rating
1. ATSDR – HazDat Database	8	8	2	18
2. Chemical Sampling Information (CSI)	6	9	2	17
3. ESIS – European Chemical Substances System	8	10	3	21
4. EXTOXNET	6	5	2	13
5. GESTIS – database on hazardous substances	8	9	5	22
6. Haz-Map	5	8	2	15
7. Hazardous Substances Data Bank (HSDB)	8	10	4	22
8. Hazardous Substances Information System (HSIS)	4	9	3	16
9. High Production Volume Information System (HPVIS)	3	6	2	11
10. IARC Monographs	3	6	4	13
11. IPCS INCHEM	6	8	4	18
12. International Toxicity Estimates for Risk Database (ITER)	5	8	3	16
13. IRIS database for risk assessment	5	10	3	18
14. MSDS Database – (Material Safety Data Sheet db): OHSAH	5	6	4	15
15. NIOSH Pocket Guide to Chemical Hazards	8	9	4	21
16. PAN Pesticides Database	4	8	4	16
17. Scorecard	3	7	4	14
18. Screening Information Data Set (SIDS) for High Volume Chemicals	4	8	4	16
19. SOLV-DB	6	4	3	13
20. The chemical database	3	8	2	13
21. TOXNET	6	11	4	21

(Maximum scores of needs = 9, quality = 13, and usability = 5. Minimum scores of needs = 2, quality = 4 and usability = 0.)

The total score for *quality *ranged from 4 to 11, with none of the databases achieving the maximum score of 13. TOXNET came closest to the maximum score with 11 points. The items that were most often missing were information on updating, peer review, and number of chemicals. The number was often hard to find and sometimes we had to calculate it ourselves. Some databases did not provide information on important quality items. It is possible that in some cases the quality was reasonable but that it was impossible to judge because the information was missing.

The total score for *ease of use *ranged from 2 to 5 with the GESTIS database scoring the maximum 5 points. Items that were most often missing were help functions, such as description of the contents of the database and/or instructions on how to use the database. The user interfaces were often found too simplified with only one or two fields to search from and with no link to user support, help function, or other important information for the user.

The following five databases fulfilled most of the criteria for user needs with a score of 8 out of 9 points: ATSDR, ESIS, GESTIS, Hazardous Substances Data Bank (HSDB), and NIOSH Pocket Guide to Chemical Substances. The quality of the information was highest for TOXNET with a score of 11 out of a maximum of 13, 10 for ESIS, HSDB and IRIS, 5 to 9 for the remaining databases.

Not all databases consist of a single entity but several actually constitute a cluster of databases such as ESIS, Haz-Map, IPCS INCHEM, and TOXNET. In our evaluation, we primarily targeted the whole entity, but from TOXNET some individual databases were also evaluated, because of being found in our systematic search.

Table 2. Comparison of the rating results of needs, quality, usability and total rating. (Maximum scores of needs = 9, quality = 13, and usability = 5. Minimum scores of needs = 2, quality = 4 and usability = 0.)

### A model for the process of finding toxicological information

Practitioners are always under time pressure to find quick answers to questions that arise from practice and thus need an efficient way for finding answers. That is why a model for how to proceed when seeking answers to practical questions is important [14]. Finding answers to questions is a step by step process, where one should first 1) define the information needs and convert them into a focused question and then 2) decide how and from where to find the answer and the best evidence.

The first step should be *checking the identity and determining the proper chemical name of the substance*. To this end specific search engines are available such as Chem ID Plus in TOXNET or eMolecules. Chem ID Plus uses fuzzy similarity searching. To find information on a chemical here, the name of a substance need not be exactly right as is the case in eMolecules. The objective is to locate the CAS or EINECS number, chemical names or synonyms, commercial names, or components of a substance to make searching efficient. After searching for a chemical, it is possible to view compound information and obtain the CAS number and possible synonyms. Those databases presented here provide mostly information on pure chemicals or pure chemical compounds that can be found by their CAS-numbers and provide much less frequently information on commercial chemical products.

The second step should be *checking Safety Data Sheets*. The chemical supplier, manufacturer, or importer should provide detailed information on the Safety Data Sheet, and organizations using chemicals should have these data sheets available. If not, we suggest using the database HSDB to check classification and labelling (data e.g. on handling, storing, or use of a chemical substance). The classification of the chemical will provide a basis for understanding the toxic profile. The datasheets contain valuable information such as R and S sentences, providing information on risks (R), safety advice (S), and Threshold Limit Values or Occupational Exposure Limit values – tools that can be immediately applied to practice. However, it is important to know that the information on the sheets is not always validated or checked [19].

The third step is *finding evaluations and toxicological profiles*. We advise consulting the databases with the highest ratings such as GESTIS, NIOSH Pocket Guide to Chemical Hazards, ESIS, or TOXNET which contains HSDB, Haz-Map and IRIS. Some databases cover particular types of chemicals. In case information on pesticides is needed, it is advisable to consult the PAN database, whereas for information on carcinogens, the best choice is IARC monographs, but only summaries of monographs are accessible free of charge. An advantageous feature of Haz-Map is that it links jobs and hazardous tasks with occupational diseases and their symptoms.

The fourth step is *finding more evidence*. In case the user finds the factual data lacking in some respects, for instance the information is not fully up-to-date, the databases include references to bibliographic databases such as Toxline, PubMed, CISDOC, NIOSHTIC-2, or Riskline, and eventually also links to original articles. This is not possible without knowing the identity of a chemical, which emphasises the importance of the first step. However, the CAS number is not used in all the articles on toxic chemicals indexed in PubMed.

**Figure 1:**
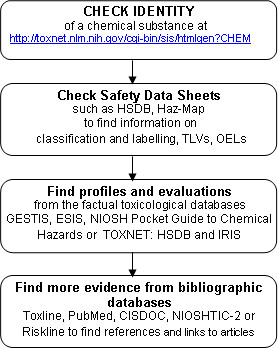
A flow chart for finding toxicological information from the databases.

The information resulting from these searches should always be critically appraised based on appropriate criteria [20]. A flow chart for locating toxicological information is presented in figure 1.

## Discussion

By means of a systematic search, we found 21 databases that met the criteria for a searchable database on toxic chemicals available free of charge on the Internet. We developed a rating system based on the needs of OHPs, and the quality and usability of the databases. In our scoring of the databases based on the rating system, the highest points (over 20) were received by five toxicological databases: ESIS, GESTIS, Hazardous Substances Data Bank (HSDB), NIOSH Pocket Guide to Chemical Hazards and TOXNET.

The GESTIS database was rated with the same criteria as the other databases. Despite the fact that not all the profiles in GESTIS are available in English, it was rated among the top databases in the study. In contrast to the other databases, predominantly in English, its main language is German.

The strength of our study is that we developed our own rating system based on the practical questions of the OHPs, quality of the information provided, and ease of use of the database. Quality of information is a critical factor in the decision making of OHPs: low quality is a known impediment in the use of information. The following relevant criteria were used to assess quality: level of information, number of chemicals covered, existence of peer review, reputation of publisher, and up-to-datedness of information. The quality judgements were based on human assessment thus entailing varying degrees of subjectivity. To increase the reliability of the evaluation and mimic the real life situations, we used several evaluators with different areas of expertise and educational backgrounds.

We also constructed a model for the search process in order to assist the OHPs in searching for information. The challenge for the OHPs is to gain adequate, valid, and relevant information efficiently. Other authors have also built comparable models, but with no clear process description [3] or with an orientation on how resources should be integrated in the future [21,22]. None of the models were targeted explicitly to OHPs.

In spite of the clear criteria established, it was not easy to judge complex databases such as ESIS, Haz-Map, INCHEM, TOXNET, and their ability to meet the users' needs and the level of quality. These databases are heterogenous, which complicates uniform interpretation when comparing and scoring contents from different sources. In general, we experienced difficulty in locating information needed to make a proper assessment because it either did not exist or was inconveniently placed, requiring many clicks in the website. Davis et al. also found the aggregate databases with information in different sites complicated and sometimes impossible to evaluate [23].

In the systematic search we found a greater number of free of charge toxicological databases aimed at OHPs than listed by Wright. He had a remarkable collection of fee and non-fee databases in his evaluation. However, the majority of databases listed by him are from fee-based suppliers [3]. Guerbet et al evaluated 22 free of charge factual databases, nine were also found by us and included in our assessment. Two TOXNET databases in their study, Chemical Carcinogenesis Research Information System and Genetic Toxicology, were not found in our systematic search and that is why we evaluated them only as part of the TOXNET cluster. However, in Guerbet's study, there were several databases which did not fulfil our inclusion criteria for a database. Two were in French, some without a search engine and others very specialized (e.g. genotoxicity or ecotoxicity) with extremely limited content [18].

In our study, we did our best to find a representative collection of databases for OHPs, but it is always possible that we have missed a database that would have met our criteria. However, when we compare our results to those of other authors, we are quite confident that we have identified all currently available databases fulfilling our inclusion criteria. On the other hand, there is a growing amount of information that remains outside the reach of Internet search engines. Efficient use of the Web requires the discovery of and familiarity with sites housing their own query engines for databases. Content or metadata of contents are stored in the databases, the search engines of which produce results dynamically in response to a direct request [4]. Moreover, the Internet is constantly changing and therefore we find that continuous evaluation and rating is needed in order to ensure that there is continued direction and information available for OHPs concerning the best and the most appropriate databases to use.

Voigt et al also used a quantitative method for quality assessment of toxic chemicals databases for two kinds of chemicals: high production volume chemicals (HPV) and pharmaceuticals. In their results, GESTIS and ESIS were ranked the highest containing information on all 12 HPV chemicals used in the test. In this assessment, HSDB was rated second best failing to provide information on one chemical used in the test. ESIS was mentioned as giving remarkably good results [9]. However, their results were not intended for use by practitioners and their assessment was not as comprehensive as ours. Guerbet et al evaluated factual databases with free access, specialized in toxicology and maintained mainly by USA organizations using 27 criteria related to physicochemical and ecotoxicological aspects and time of environmental half-life. In this evaluation, HSDB was found the most efficient database, and it was recommended for a general search for information about any chemical [18].

Weiss reminds that peer reviewed datasheets and articles contain information of the highest quality [24]. Despite the importance of peer review for the credibility of scientific products, there is no consistent way of announcing it. Peer review is one of the procedures used to ensure that the quality of published information meets the standards of the scientific community. A peer review process should be transparent by making available in the Internet pages the written evidence such as peer reviewers' names, the agency and potential conflicts of interest of producers and providers of information [25]. Some Internet sites, especially online journals have instituted peer review processes, but unless a site clearly indicates that it has been reviewed, it is safer to assume otherwise [13]. It was difficult to find any information on peer review on many of the sites housing the databases.

Emerging Internet technology opens up totally new possibilities in aggregating information about toxic chemicals from different sources. Yang reviewed the various efforts that are currently underway to construct new types of toxicological databases including standardization of the content of information [22]. Mendonça et al describe the tools that could facilitate access to, extraction of, and summarization of information needed by clinicians in their practice. The study is about the development of informatics infrastructure for evidence based practice [21]. Revere also draws attention to the development of online information resources, but reminds her readers about the diversity of the public health workforce [15]. OHPs have different needs of information which should be considered in the development of the contents and interfaces of databases.

In Europe the new chemicals regulation, REACH, will introduce new information in the near future. It will generate information of substances, properties, classification, and labelling, which will be made available in a main database. The European Chemicals Agency (ECHA) which started in Helsinki on 1 June 2007 is responsible for the provision of these data via Internet [17]. It is our hope that this process will go beyond the formation of another database and will produce a co-ordinated activity with other providers of toxicological information that will overcome the problems that we have reported.

## Conclusion

The Internet provides toxicological information that can be used to support practical decision making of occupational health professionals. There is a need to improve access to credible and reliable toxicological information and to enhance the decision-making process by developing tools for the evaluation of the available information. The producers of databases must become more familiar with the users and target their databases increasingly to specific audiences such as OHPs. Reliability combined with intelligibility and coverage of information are key factors for users.

## Competing interests

This manuscript was produced without any sponsoring. The authors declare that there are no competing interests.

## Authors' contributions

IL and JV developed the idea for the study, IL developed the scoring system and carried out the search, GF contributed in the design of users needs, MLu, ML, IL and JV assessed the contents and the quality of the databases, all authors commented on the plan and various drafts of the manuscript.

**Table 3:** The rating scale

**User needs**	**Rating scale**	**Score**
Chemical name, CAS, synonyms, trade names etc.	advanced search + CAS and names have own fields or a drag-down menu or a list to choose a name from	3
	advanced search, a search field plus another field for names or a drag down menu or a list to choose a name from	2
	simple search, one field for all searching	1
Health hazards (carcinogenicity, mutagenicity, teratogenicity, endocrine modification, irritation etc.)	detailed description and analysis	2
	very short description (fairly narrow, summarizing and involving the key points)	1
Exposure routes	yes, routes mentioned	1
	not at all mentioned	0
Methods for measuring exposure	yes	1
	not found	0
Exposure limits such as TLVs or OELs mentioned	yes	1
	not found	0
Preventive measures	yes	1
	not found	0

**Quality of contents**	**Rating scale**	**Score**

Topics covered	wider topic selection	2
	specialization, one topic such as pesticides or cancer	1
Level of information	analysed information: Pragmatic scientific or technical reporting: Scientific proofs of evidence, a growing body of sources used to prove toxicity and to assess the risks of the chemicals to human health. Includes necessary details and subject areas as well as examples of risk assessments	3
	analysed information: same as above, but does not include risk assessments	2
	general: unambiguous, definitive and easily interpreted. Extrapolate scientific findings or expert opinions to the wider public in an easy to understand form	1
Number of chemicals	10 000 to 100 000 chemicals	3
	1000 to 9 999 chemicals	2
	less than 999 chemicals	1
Peer reviewers' names, and/or the publisher's response to the peer reviewers' report(s) available.	evaluation of information by a scientific committee or special peer reviewers	1
	no information on peer review	0
Reputation of publisher of the database	regulator, policy maker, national or international organization	2
	university or other parties	1
Up-to-date	information on update	1
	no information on update	0

**Usability**	**Rating scale**	**Score**

Ease of use	usable at one glance	2
	takes time, use requires help texts	1
	the database is arduous, one use cycle not enough	0
Help available	information on both contents and the use of the database	2
	information either on contents or how to use the database	1
	no information on contents or how to use the database	0
Language	contents also in other languages	1
	English only	0

**Table 4:** All inclusive results of the ratings of the user needs, quality, and usability factors

	**User needs**						**Quality of contents**						**Usability**		
**Database name**	**Chem. name**	**Health hazard**	**Expos. route**	**Meas. expos.**	**Limit values**	**Prev. meas.**	**Topics**	**Level infor.**	**Numb. chem.**	**Peer rev.**	**Reput.**	**Freq.**	**Navig.**	**Help availab.**	Lang. **)

1. ATSDR-HazDat	3	2	1	1	1	0	2	2	1	1	2	0	1	0	1
2. Chemical Sampling Information (CSI)	3	1	0	1	1	0	2	1	2	1	2	1	2	0	0
3. ESIS	3	2	1	1	1	0	2	2	3	1	2	0	1	2	0
4. EXTOXNET	1	1	1	1	1	1	1	2	1	0	1	0	1	1	0
5. GESTIS	3	2	1	0	1	1	2	3	2	0	1	1	2	2	1
6. Haz-Map	2	2	0	0	1	0	2	1	2	0	2	1	2	0	0
7. Hazardous Substances Data Bank (HSDB)	2	2	1	1	1	1	2	2	2	1	2	1	2	2	0
8. HSIS = Hazardous Substances Information System	2	1	0	0	1	0	2	2	2	0	2	1	2	1	0
9. High Production Volume Information System (HPVIS)	1	1	0	1	0	0	2	1	0	0	2	1	1	0	1
10. IARC Monographs ***)	1	1	1	0	0	0	1	2	1	0	2	0	2	1	1
11. IPCS INCHEM	2	1	1	0	1	1	2	3	*)	1	2	0	2	2	0
12. International Toxicity Estimates for Risk Database (ITER)	2	1	1	0	1	0	2	2	1	1	1	1	2	1	0
13. IRIS database for risk assessment	2	2	1	0	0	0	2	2	2	1	2	1	1	2	0
14. MSDS Database	1	1	1	0	1	1	2	2	*)	0	1	1	2	2	0
15. NIOSH Pocket Guide to Chemical Hazards	3	1	1	1	1	1	2	2	1	1	2	1	2	2	0
16. PAN Pesticides Database	2	1	1	0	0	0	1	2	3	0	1	1	2	2	0
17. Scorecard	2	1	0	0	0	0	2	1	3	0	1	0	2	2	0
18. Screening Information Data Set (SIDS) for High Volume Chemicals	2	1	1	0	0	0	1	2	1	1	2	1	2	2	0
19. SOLV-DB	3	1	1	0	1	0	1	1	1	0	1	0	2	1	0
20. The chemical database	1	1	1	0	0	0	2	1	3	0	1	1	2	0	0
21. TOXNET	1	1	1	1	1	1	2	2	3	1	2	1	2	2	0

*) no information found

**) Chem. name = Chemical name; Expos. route = Exposure route; Meas. expos. = Measure exposure; Prev. meas. = Preventive measures; Level infor. = Level of information; Numb. chem. = Number of chemicals; Peer rev. = Peer review; Reput. = Reputation; Freq. = Frequency; Navig. = Navigation; Help availab. = Help available; Lang. = Language

***) only summaries are available free

**Table 5:** All databases found; also excluded databases listed

**Database**	**Contents description**	**Reason for inclusion/exclusion**	
ATSDR – HazDat Database-	information to prevent harmful exposures and diseases related to toxic substances	yes, fulfils the criteria	1.
Australian Occupational Health and Safety Index		appears not to be working	
CDC – topic page on chemicals	chemicals – one of the occupational health and safety topics	not a database, no search engine	
CHE disease and toxicant database	summarizes links between chemical contaminants and approximately 180 human diseases or conditions	not a factual database	
Chemfinder	a free search by chemical name, CAS number, molecular formula or weight	not enough factual data, suitable for CAS search etc.	
Chemical Sampling Information (CSI)	a large number of chemical substances that may be encountered in industrial hygiene investigations	yes, fulfils the criteria	2.
CISDOC	information about occupational safety and health publications, including summaries of their content	not a factual database	
ESIS	Existing Commercial Substances, European List of Notified Chemical Substances, High Production Volume and Low Production Volume Chemicals, Classification and Labelling, Chemical Data Sheets etc.	yes, fulfils the criteria	3.
EXTOXNET	information on pesticides – written for the non-expert	yes, fulfils the criteria	4.
GESTIS – database on hazardous substances	for the safe handling of chemical substances at work, e.g. health effects, necessary protective measures in case of danger (incl. first aid).	yes, fulfils the criteria	5.
Haz-Map	links jobs and hazardous tasks with occupational diseases and their symptoms	yes, fulfils the criteria	6.
Hazardous Substances Data Bank (HSDB)	focuses on the toxicology of potentially hazardous chemicals; also offers information on human exposures, industrial hygiene, emergency handling procedures, environmental fate, regulatory requirements, and related areas.	yes, fulfils the criteria	7.
HSIS = Hazardous Substances Information System	allows you to find information on hazardous substances that have been classified in accordance with the Approved Criteria for Classifying Hazardous Substances [NOHSC:1008(2004] 3rd Edition and/or have National Exposure Standards declared	yes, fulfils the criteria	8.
High Production Volume Information System (HPVIS)	access to select health and environmental effect information on chemicals that are manufactured in exceptionally large amounts	yes, fulfils the criteria	9.
IARC Monographs	helps to identify environmental factors that can increase the risk of human cancer. These include chemicals, complex mixtures, occupational exposures, physical and biological agents, and lifestyle factors.	yes, fulfils the criteria	10.
ILO Encyclopaedia	comprehensive and accurate coverage of the core allied fields encompassing occupational health and safety. ILO Encyclopaedia is an important source. It is available both for fee and free of charge. Site we found was not free and we did not accept it for scoring. There was no cause for changing our strategy.	not, was not free	.
IPCS INCHEM	information on commonly and globally used chemicals that may also occur as contaminants in the environment and food	yes, fulfils the criteria	11.
International Toxicity Estimates for Risk Database	human health risk values and cancer classifications for over 600 chemicals of environmental concern from multiple organizations worldwide	yes, fulfils the criteria	12.
IRIS database for risk assessment	human health effects that may result from exposure to various substances found in the environment	yes, fulfils the criteria	13.
MSDS Database: OHSAH	province-wide Workplace Hazardous Materials Information System (WHMIS) needs.	yes, fulfils the criteria	14.
MSDS Databases: Cornell University	contains 140 000 MSDSs. The DLA (Defense Logistics Agency) developed HMIS (Hazard Material Information System) to track and make available the MSDSs the government processes annually. Collection of databases.	whole database is not free of charge	.
NIOSH Pocket Guide to Chemical Hazards	several hundred chemicals/classes found in the work environment.	yes, fulfils the criteria	15.
NIOSHTIC-2	a bibliographic database of occupational safety and health publications, documents, grant reports, and other communication products	not a factual database	
OECD Database of Risk Assessment Models	models (computerized or susceptible to computerization) used by OECD Member governments and industry to predict health or environmental effects (e.g., QSARs), exposure potential, and possible risks.	does not fulfil the criteria	.
PAN Pesticides Database	current toxicity and regulatory information for pesticides.	yes, fulfil the criteria	16.
Pesticides	Evaluation documents are the papers presented to the ACP at the meeting with only the commercial secrets withdrawn.	not, does not fulfil the criteria	
Riskline	bibliographic database of both environment and health Important tool in risk reduction programs.	not a factual database	
Scorecard	provides detailed information on more than 11,200 chemicals	yes, fulfils the criteria	17.
Screening Information Data Set (SIDS) for High Volume Chemicals	health and environmental risk assessment of chemicals.	yes, fulfils the criteria	18.
SOLV-DB	solvents data.	yes, fulfils the criteria	19.
The Association of Occupational & Environmental Clinics: AOEC Exposure Codes	exposure coding system	not a factual database	
The chemical database	25 496 hazardous chemicals or 'generic' entries, information compiled by the author from a large number of sources.	yes, fulfils the criteria	20.
TOXNET	databases on toxicology, hazardous chemicals, environmental health, and toxic releases	yes, fulfils the criteria	21.
